# Appraisal of Observance of Behaviour Change Communication Programme for Maternal and Child Health at First Level of Midwifery Practice in Kaduna State Nigeria

**DOI:** 10.5812/nms.11361

**Published:** 2013-09-15

**Authors:** Bridget Omowumi Akin-Otiko, Busisiwe Rosemary Bhengu

**Affiliations:** 1 School of Nursing and Public Health, University of KwaZulu-Natal, Durban, South Africa

**Keywords:** Behaviour change communication, Integrative framework, Midwife, Appraisal

## Abstract

**Background:**

Behaviour Change Communication (BCC) is a key component of the roadmap adopted by the Nigerian government to address the high maternal and child mortality in the country.

**Objectives:**

The purpose of the study was to appraise the participation of midwives in BCC at the first level of health care in Kaduna State, Nigeria before planning a context specific and sustainable BCC capacity building programme.

**Materials and Methods:**

In-depth interviews were conducted with nine midwives selected by maximum variation technique across Kaduna State. Content analysis of the interviews was performed using *a priory codes *derived from the integrative framework.

**Results:**

The integrative framework provided a comprehensive appraisal of BCC in the facilities. Health talks were unplanned, difficult and more task-oriented than being behaviour change focused. The required skills, integrated services to enhance behaviour change by clients, and enabling environment, were missing. The findings were used, in collaboration with the midwives to develop and implement a context specific and efficient capacity building programme.

**Conclusions:**

The framework was adequate in identifying the gaps in the BCC activities of midwives at the facilities. There is a need to understand and support midwives with their BCC activities. Government policies should be brought closer to frontline staff who would implement them, by engaging such staff all through the process of developing the policies.

## 1. Background

Lack of access to appropriate information and services to encourage desired health behaviours is one of the major barriers to achieve maternal and child health related Millennium Development Goals (MDGs) in Nigeria (1). Behaviour Change Communication (BCC) was therefore, incorporated into the country’s reproductive health policyto* accelerate the attainment of the MDGs *(1). BCC was expected to improve knowledge, create positive attitudes, and promote satisfactory health behaviour, including increased utilization of maternal, new-born, and child health (MNCH) care services (1). *In this study, *BCC referred to midwives using appropriate interpersonal communication and counselling (IPCC) skills within a supportive environment to enhance the knowledge, attitudes, practices and skills of their clients for maternal, new-born and child health, and improved utilization of available services within the facilities (2). At the health system level of BCC programme, the strategic approach is both *service oriented* and* interpersonal communication focused * (1). The *service oriented strategy* included ‘improving the image of the service providers for increased client confidence; holding health talks or sessions at the clinics; and creating opportunities for integrated service approach’. The *interpersonal communication strategy *involved ‘training health workers to enhance their skills on interpersonal communication and counselling (IPCC)’ to make available to clients, required information and support, to affect appropriate lifestyle changes, promote their health, and enhance their utilization of services (1).

Although midwives accept client education as their major responsibility, (3) studies show that they lacked the IPCC skills to facilitate the required behaviour change by consumers (4, 5). Researchers observed that midwives found educating clients challenging (3) though they still gave health talks routinely (3, 6). They attributed the difficulty to inadequate training (7), clients’ culture (8), poverty and poor level of education (9), as well as staff shortage and heavy workload (10). Other documented militating factors included lack of appropriate job aids and guidelines (7), external interruptions, lack of privacy, and inappropriate conversation environment (2, 9, 11)*.* IPCC skills training must be appropriate to the specific context, considering both midwives and their clients (10) ; hence, to plan and implement a context specific and relevant capacity building programme for midwives in Kaduna State, it was necessary to appraise the current BCC activities by the midwives.

**Figure 1.**
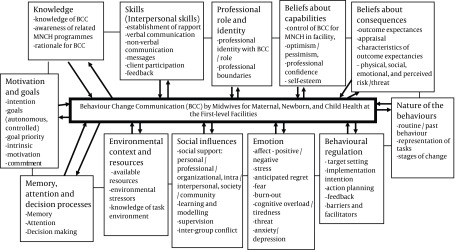
Diagrammatic representation of the twelve domains in Michie et al’s (2005) Integrative Framework for Studying the Implementation of Evidence Based Practice applied to Midwives’ Behaviour Change Communication for MNCH. Weaknesses in the domains could hinder BCC activities (indicated by arrow pointing to the centre box), while contextually relevant capacity building programmes to address the weaknesses, and implementation of feasible local action plan could affect the domains positively and make BCC for MNCH result-oriented and effective (indicated by arrows pointing away from the centre box to the domains).

According to Michie et al. ( 12 ), health professionals would not execute government programmes if the underlining factors that have implication for their compliance are not adequately identified and attended to. Michie et al.’s ( 12 ) *Integrative Framework for Studying the Implementation of Evidence Based Practice *has twelve clearly defined and observable domains. The domains could be manipulated, as required of any theory to be applied in behaviour change intervention ( 13 ). The twelve domains are:

(i) knowledge,
(ii) skills,
(iii) social/professional role and identity,
(iv) beliefs about capabilities,
(v) beliefs about consequences,
(vi) motivation and goals,
(vii) memory, attention and decision processes,
(viii) environmental context and resources,
(ix) social influences,
(x) emotion,
(xi) behavioural regulation,
(xii) nature of the behaviours. Each domain consists of ‘a set of similar theoretical constructs’ (Figure 1).

By deciding to use this integrative framework, we assumed that:

1. The twelve domains would reveal the strengths and weakness related to midwives’ behaviour change communication for MNCH in the facilities.
2. Clear and comprehensive identification of the weaknesses using the twelve domains would simplify the process of identifying appropriate strategies to address hindrances to BCC activities in the health facilities.
3. If the weaknesses were correctly identified and the suggested solutions were appropriate, then the intervention would be successful.

## 2. Objective

The purpose of this study was to appraise the participation of midwives in BCC at the first level of health care in Kaduna State, Nigeria before planning a context specific and sustainable BCC capacity building programme.

## 3. Materials and Methods

Approval to undertake the study was obtained from the University of KwaZulu-Natal, South Africa, and Kaduna State Ministries of Health and Local Government. A qualitative approach was considered suitable for the study due to our interest in the experiences of midwives in their workplace settings (14). First level maternal and child care is provided in primary health care facilities and rural hospitals by midwives, where midwives are available. The maximum variation technique which considers the criteria of interest to the study, and the possible variations and similarities in the population, was used to recruit the midwives (15). A total of nine key-informant female midwives were recruited from nine facilities in eight Local Government Areas (LGAs) across the northern, southern, and the central senatorial zones of Kaduna State. This was in line with the interest of the State Government that participants be spread across the state as much as possible. Seven of the facilities were primary health care facilities and the other two were rural hospitals. Six of the facilities were from rural settings, one from urban setting and two from urban slum. The choice of in-depth interview (IDI) method of data collection was in view of the shortage of midwives in the State, and the possibility of not having an adequate number of midwives in any of the health facilities to constitute an acceptable minimum number for a focus group discussion.

Along with the integrative framework, Michie et al. (12) provided model questions to guide researchers in conducting investigative interviews for example: in the *Knowledge domain *-**“Do they know about X?”; in the *skills domain *- “How easy or difficult do they find performing X to the required standard in the required context?”; in the *Beliefs about consequences domain *- What do they think would happen if they do X? What do they think would happen if they do not do X? (Prompts); and in the *Nature of the behaviours domain *“Who needs to do what differently when, where, how, how often and with whom? How long are changes going to take? Are there systems for maintaining long term change?”, “How do they know whether the behaviour has happened?” The questions were adapted to develop the structured IDI guide used for interviewing the midwives. In its adapted form, the instrument consisted of questions about BCC by midwives in the facilities instead of ‘X’ in the guide.

### 3.1. Ethical Considerations

This study was approved by the institutional review board and the ethics committee in our university. Written informed consent was obtained from each midwife, to participate in the study, to allow an in-depth interview to be conducted, and also for the interview to be tape-recorded (16). The data collection lasted from Monday 9^th^ to Friday 23^rd^ November 2009. Each midwife was interviewed personally by B.O. (the first author). The interviews were tape-recorded. A total of nine IDIs were conducted with each interview lasting an average duration of 15 minutes.

### 3.2. Data Analysis

B.O. transcribed the audio recordings. Simple content analysis recommended for descriptive qualitative studies was performed (14). *A priori *codes were created (17) using the twelve theoretical domains. Words, phrases and sentences related to the codes in the texts were identified and highlighted. Data matrices were created manually (18). Highlighted items from each IDI text were retrieved and arranged under each domain within the matrices, participant by participant; for analysis and interpretation (17).

Credibility and dependability were ensured through(14) facilitated openness, detailed daily diary of events maintained by the researcher, and voice recording of interviews. *Authenticity* was by providing direct quotes from the midwives. Confirmability was through presentation of the data matrices to the midwives at a three day search conference held on 27th – 29th January 2010. At the conference, the midwives verified the data (19), appraised the BCC activities for maternal, new-born and child health in the facilities, and decided on appropriate intervention. Theoretical connection of the study through the adaptation of Michie et al.’s (12) theoretical domains and the Federal Ministry of Health’s BCC strategy, strengthened it and could enhance transferability (15).

## 4. Results

Practising midwives in the State are females so all the midwives were females. Four of the midwives served in the mixed central zone of the State, two served in the predominantly Muslim northern zone, and three in the predominantly Christian southern zone. Six of them were Christians while the other three were Muslims. Age ranged between 39 and 59 years (mean 46 years). Eight of them were married and one widowed. Seven were of Kaduna State origin and two were from other states in the country. They had practised midwifery for 12 – 30 years (mean 22 years).

Two midwives explained BCC for maternal, new-born and child health (MNCH) “I think it’s a process whereby health workers are being informed on the way that they should be more friendly with the clients, and they change their attitude (MF2 PHC urban slum) “What I understand is that it’s a process… a way to change the behaviour of people, in a certain community or certain belief, … or in terms of health in terms of either the mother or the child” (MF6 PHC urban slum). The other seven had not heard of BCC for MNCH and explained it as the health talks given during antenatal clinics “I have no idea” (after prompt) “It is done in form of health talk” (MF4 PHC urban).

Some of the midwives believed they had the required skills for BCC and only one confessed “Well, the new methods I don’t know” (MF7 PHC rural). One of the two midwives who explained what the BCC plan was about, requested a uniform manual or protocol to guide BCC for MNCH activities in all the health facilities, saying: ‘In future could you produce something standard so that all health facilities would be using it in common, so that everybody would have a book, balanced, the information sound and accurate on different levels?’ (MF2, PHC urban slum).

The midwives recognized BCC for MNCH as their professional responsibility and did not see it as the exclusive responsibility of midwives; “It’s our work” (WF5 PHC Rural). “If we keep things to ourselves, we can’t do everything” (WF7 PHC rural) “Like these CHEW in local government and mainly doctors, nurses, midwives in hospital do it” (WF8 Rural Hospital). The midwives pointed that the BCC for MNCH guidelines could improve the attitude and practice of midwives.

Most of the midwives felt what they were currently doing in the form of health talks, is difficult. ‘Ha! It’s not easy. It’s not easy (shakes her head). That’s the thing’ (MF6, PHC urban slum). Optimistically, they said that repeating health talks, allowing women ask questions, and, continuing to try, would solve the problem. None of them had the capability to maintain the required standard for BCC; to some, that depended on what the authority in-charge of the facility allows.

The midwives believed that BCC was good and could produce some positive changes in the knowledge, attitude and behaviour of the clients, and there would be improvement in the health of both mother and child. The cost of doing BCC for MNCH, according to the midwives was cheaper than that of not doing it, because, there would be deaths of mothers and children if there was no BCC. MF1 (PHC rural) said, ‘They are ignorant of everything, so there are dangers; most of them would have problems, most of their children would die, even the women’. Most of the midwives felt good and satisfied when they educated clients due to the advantages, and MF6 (PHC urban slum) indicated that she felt she had “not achieved something” any day she failed to educate clients.

Most of the midwives were eager and happy to do BCC for MNCH, “I always feel eager to do it. Any day it’s my antenatal day I’m very, very happy.” (MF5 PHC rural); “I’m eager to do it because I want everybody to have the knowledge” (MF8 Rural hospital). Others were not so eager to do it due to fear from people culture and religion, and because some of the women might not comply. Regarding fear, MF4 in urban PHC said “*Hen*!, we still tell them because if we don’t tell them, in fact you would face problem, we are the ones suffering it”; because many women would come in with complications and without the required things to take the delivery and care for the baby. Regarding environmental barriers, some midwives still gave the health talk routinely even if not in details or for the required length of time. What encouraged the midwives included: their belief in God as their rewarded, women’s freedom to discuss with them, women’s improved knowledge, and positive changes in the community which could be credited to them. “For you to do something may be in one of the villages or community where you are working, and you are able to change their attitude at least, they say since this woman came, the hospital has changed, I am happy”, (MF3 PHC rural).

The health talks were given at antenatal clinics daily in some facilities, and once or twice weekly in others. The midwives remembered to give health talks for various reasons, which included the need for it, and their having something to say. They decided not to do it sometimes when results of doing it were not visible. According to MF6 (PHC urban slum), ‘When some don’t want to hear; after you talk, talk, talk, talk, and they refuse, you say Hun! Why am I bothering myself? Let me forget about them’.

The gross shortage of midwives, small space for antenatal clinic, lack of conveniences, lack of privacy and safety, and unresolved inter-professional issues, hindered BCC for MNCH in the facilities. Competing tasks such as an emergency, heavy workload with too many patients and registering new pregnancies interfered with health talks. According to the midwives, interacting with clients was time consuming. MF4 (PHC urban) took a deep breath and said,**‘Well, it’s only taking time. The time you spend, you continue to attend to one person and tell them individually, because it’s during booking that we always do that, we interview individually, and we interact with them’. When the midwives had to spend a long time with a particular client other clients felt neglected and complained. Some of the facilities had posters and the urban facility had promotional materials and information leaflets from some companies. In one of the facilities, the posters were put behind the door due to lack of space. There were no job aids in some facilities. MF5 (PHC rural) said, ‘We don’t have anything; I only use my initiative to tell them the most important things I know’.

MF3 (PHC rural) enjoyed the support of the health committee in her community, while others did not. Some used their personal money to buy items to motivate women. The support expected or received, depended on the type of community, the type of religion, or the behaviour of interest vis-à-vis the people’s culture. Many of the midwives associated the difficulties they had with BCC for MNCH with the clients’ socio-cultural background, poverty, low level of education, and language barrier. The midwives felt that some women did not listen to or comply with what they were told, and described women as being difficult to convince, slow of learning, and sometimes saying they were charmed (bewitched) rather than accepting their diagnosis. Some women discriminated against the midwives. According to MF7 (PHC rural) ‘They don’t even take you seriously, because you are not from their community, not a part and parcel of them’.**The midwives expressed strong desire to learn more about BCC for MNCH, and believed they would gain from other people.

BCC for MNCH evoked pleasant emotions in the midwives, especially when clients complied with the counsel and experienced positive changes. To MF4 (PHC urban) BCC provided opportunity for a “humanitarian job”. The midwives felt that their negative emotions should be controlled and not be allowed to interfere with BCC. MF7 (PHC rural) who was not happy with the way a crisis in her workplace was handled by her supervisor said, “Sometimes I feel bad doing it; true! Unless the existing crisis was resolved …” 

According to MF6 (PHC urban slum) “You prepare because some patients would say some words that would be hurting to you but you should be prepared to them, be patient with them and take it, do not be angry, she may ask you questions and if you are not prepared you would be stranded” The other midwives believed that no preparation was needed before giving health talks since they had been doing it before. They did not also document whatever happened during the talk or counselling sessions.

What the midwives were currently doing was mainly giving health talks to pregnant women in groups, and occasional counselling on demand or when deemed necessary. They saw the health talks as BCC. Other elements of the service oriented strategy were unknown to the midwives. Services were only on scheduled days and time. To implement BCC according to the required standard some of the midwives wanted bigger and better place with adequate privacy; many needed posters wanted government to provide incentives to motivate mothers. According to the midwives, to know if BCC for MNCH was taking place in a facility, midwives needed to be supervised, and the monitoring and evaluation data reviewed. Most of the midwives felt only their supervisors could make changes possible MF9 (Rural hospital) said, “You see I cannot answer this question it’s (laughing) depending on ourselves (laughing, referring to the supervisor). Hen en, (laughing) if you see him he would know how to …”; but she believed that if changes were effected midwives would sustain them “We can sustain it”.

The accuracy of the data in the matrices was confirmed by the midwives at the three day conference. The midwives identified the strengths and weaknesses in BCC for MNCH at the facilities from the data, and proffered strategies to address the weaknesses. The comprehensive recommendations were later used to plan the capacity building programme in collaboration with the midwives. The midwives in their groups indicated in the post conference evaluation that “The workshop was very interactive, educative, and inspiring, we are motivated to improve in our services. We look forward to be trained on better ways to deliver services.” (Group 1) “It was good to attend a workshop that was related to our field of work which would help the society. Government should always organize this for their employees by emulating this.” (Group 2).

## 5. Discussion

Both the service oriented and IPCC focused strategies of the BCC framework were poorly implemented in all the facilities. Midwives lacked capability for BCC for MNCH at the facilities, though they indicated that BCC for MNCH is essential for maternal and child survival, and it is midwives’ responsibility. Midwives’ lack of IPCC skills to facilitate behaviour change by consumers, as observed in this study, is widely reported by researchers (4, 5). As in other studies, the midwives acknowledged client education was considered as a major responsibility (3). As in this study, Freda (3) also observed that educating clients was challenging. Contrary to observations in some studies that attributed the difficulty to inadequate training (7), the midwives related it to the socio-cultural background of the clients, poverty, and the clients’ low level of education. De Negri et al.(9) also documented the negative effect of poverty and poor level of education on provider-client interactions. The perceived mental and financial incapability of clients by midwives made them decide not to educate clients sometimes (20). The desire of the midwives to learn more, differed from previous reports that providers were often not interested (9). 

The belief that BCC could have positive MNCH outcomes is in line with previous findings that client education increased the knowledge of women enabling them to seek appropriate care and take necessary actions about their health (21). As documented by Storey et al. (22), the midwives were also encouraged by their clients’ improvement. Staff shortage and heavy workload (10), also interfered with BCC greatly; however, as reported by Dykes (6) and Freda (3), the midwives still arranged to give the health talks routinely. As a globally accepted component of antenatal care (20) midwives remembered to educate their clients particularly in pregnancy.

Demotivation of the midwives by the fear of religion and cultural factors is because the behaviour is rooted in culture and religion, which are volatile issues in Nigeria. Expectedly, this poses challenges to midwives, especially in rural settings (8). The request for standard messages, and the absence of posters, suggested inadequacy of, or the lack of access to, job aids by midwives. Haruna et al. (7) observed that lack of appropriate job aids and guidelines militated against client education, and limited the information provided by providers to their personal experiences as also indicated by one of the midwives in this study.

Unlike in previous reports where providers did not plan client education due to their heavy workload (23), the midwives in this study did not prepare their health talks because they felt they were experienced; whereas, preparation makes messages client-centred (24). The only element of the service oriented BCC strategy in the facilities was the routine health talks to pregnant women in groups and the occasional individual counselling. Other items such as improved provider image and the integrated service that could encourage utilization were not mentioned. Women in Nigeria did not access health care for various reasons including not wanting to go to the facilities alone (25); therefore, if women appeared eventually, sometimes in groups, it would be discouraging to send them back for coming outside the scheduled time or day; hence, the need for integrated services.

Studies support the midwives’ request for adequate accommodation and privacy, indicating that external interruptions, lack of privacy, and inappropriate conversation environment interfered with client-provider interactions (9, 11). The essential combination of adequate skills and enabling work environment for providers to be motivated to provide appropriate information in the rural areas was included in the BCC plan (1) and supported in the literature (26).

The belief of the midwives that only their superiors could initiate changes is not new; Pettersson et al. (27) also recorded similar sense of powerlessness by midwives towards initiating changes. The midwives in the current study reviewed the data on their performance, and determined the solution. It seems that, modification in the behaviour of care providers is possible when they are involved at every stage of the programming cycle. To initiate desired changes in their facilities, the midwives recommended strategies focused on the community, the government, and the midwives. Previous studies have also emphasized the need to focus on both the workers, organizations, social and environmental factors, for interventions to produce positive results (26). 

Development of midwives’ capacity to provide appropriate information and support their clients through with the latter’s behaviour change process (23) depends on relevant and sustainable capacity building programme involving midwives in the need identification.
